# Fabrication of Biomimetic 2D Nanostructures through Irradiation of Stainless Steel Surfaces with Double Femtosecond Pulses

**DOI:** 10.3390/nano12040623

**Published:** 2022-02-12

**Authors:** Matina Vlahou, Fotis Fraggelakis, Phanee Manganas, George D. Tsibidis, Anthi Ranella, Emmanuel Stratakis

**Affiliations:** 1 Institute of Electronic Structure and Laser (IESL), Foundation for Research and Technology (FORTH), N. Plastira 100, Vassilika Vouton, 70013 Heraklion, Crete, Greece; matinav@iesl.forth.gr (M.V.); pmangana@iesl.forth.gr (P.M.); tsibidis@iesl.forth.gr (G.D.T.); ranthi@iesl.forth.gr (A.R.); 2 Department of Physics, University of Crete, 71003 Heraklion, Crete, Greece

**Keywords:** 2D-LIPSS, 2D-HSFL, nanostructures, double pulse irradiation, femtosecond laser texturing

## Abstract

Femtosecond laser induced changes on the topography of stainless steel with double pulses is investigated to reveal the role of parameters such as the fluence, the energy dose and the interpulse delay on the features of the produced patterns. Our results indicate that short pulse separation (Δτ = 5 ps) favors the formation of 2D Low Spatially Frequency Laser Induced Periodic Surface Structures (LSFL) while longer interpulse delays (Δτ = 20 ps) lead to 2D High Spatially Frequency LIPSS (HSFL). The detailed investigation is complemented with an analysis of the produced surface patterns and characterization of their wetting and cell-adhesion properties. A correlation between the surface roughness and the contact angle is presented which confirms that topographies of variable roughness and complexity exhibit different wetting properties. Furthermore, our analysis indicates that patterns with different spatial characteristics demonstrate variable cell adhesion response which suggests that the methodology can be used as a strategy towards the fabrication of tailored surfaces for the development of functional implants.

## 1. Introduction

Over the past decades, laser patterning of various materials with ultrashort pulses is of particular importance due to its applicability in a vast number of areas in science, technology and industry [1,2,3,4,5,6]. The capability to produce an abundance of complex bioinspired surfaces exhibiting hierarchical structuring at length scales ranging from hundreds of nanometers to several micrometers unveils the advantage of the employment of laser technology. Numerous examples of functional surfaces have been reported on biological systems and reproduced through laser techniques. Using lasers, the fabrication of surfaces with impressive superhydrophobic [3], antifouling [7], antireflective [8], antibacterial [9], drag reduction [10] and other properties have been reported (see also [1] and references therein).

One dominant laser-based methodology to produce such surface patterns is through a self-organization fashion, and more specifically, the fabrication of the Laser Induced Periodic Surface Structures (LIPSS) (see [1] and references therein). Tailoring of the produced topographies can be achieved through varying a range of laser parameters such as the fluence, energy dose, polarization, laser wavelength, incident angle, pulse duration. Trains of linearly polarized pulses are used to generate LIPSS with periods close to the laser wavelength that are termed as Low Spatial Frequency LIPSS (LSFL) and are single axis symmetry (1D-LIPSS). On the other hand, polarization modification allows the generation of structures with multi-axial symmetry (2D-LIPSS) such as honeycomb [11] and shark skin like structures [12].

Temporally separated laser pulses have been also employed to enhance micro/nanoscale material processing capabilities through a control of the optical energy distribution and thermal effects. In previous works, it has been reported that the spatial distribution of heat can be modulated by varying the pulse separation, energy ratio and polarization states of the constituent pulses [13,14] which allows further control over the resulting surface pattern features. More specifically, apart from a variation of the area and periodicity in the LSFL covered region [14,15,16,17] it has been shown that double pulse irradiation is capable of increasing the control over the induced morphology, enabling the generation of hybrid structures, such as a mixture of LSFL and High Spatial Period LIPSS (HSFL) structures having hierarchical configuration [18] or 2D-HSFL [19,20]. HSFL structures have sizes smaller than *λ_L_*/2 (*λ_L_* stands for the laser wavelength) and they are formed at lower fluences than ripples [15]. Furthermore, unlike LSFL structures, their size is independent of *λ_L_* while it varies with laser parameters such as the fluence, pulse duration, and energy dose [21]. Their formation mechanism is still debated and the most dominant theories propose that potential generation mechanisms could involve convection flow patterns [22,23], surface tension gradients [24] or near field enhancement [25]. In previous reports it has been demonstrated that double pulse irradiation constitutes a powerful tool for controlling HSFL structure formation [23] giving rise to nanoscale biomimetic morphologies [26]. It has also been shown that delays in the picosecond timescale are suitable for the generation of HSFL structures [23,26] and the features can be controlled by varying the average number of pulses or fluence as well as the pulse separation.

Given the capability to fabricate an abundance of patterns of enhanced complexity, laser induced topographies have been broadly used as platforms to control cell behavior, as the fabrication of nano-scale patterns can differentially influence cellular adhesion and proliferation. Commonly used metallic implant materials include Ti and Ti-based alloys, which have been extensively explored [27,28,29,30] and stainless steel, which has yet to be fully investigated [31,32]. Ti is relatively well integrated into medical applications, while stainless steel can still display biocompatibility issues, mainly related to infections and osseointegration. However, as the two materials offer different mechanical properties, they are constantly being assessed for applications in different anatomic locations. Results in previous studies indicate that by fabricating different surface structures on metal surfaces, cellular adhesion and morphology can be tailored; however, this functionality is dependent on the types of cells used for testing [30].

Due to the apparent significance of the topography’s complexity, symmetry and roughness to exhibit certain properties, it is of particular importance to perform a detailed investigation of the laser conditions that lead to different biomimetic structures. Given the impact of double pulse irradiation on the fabrication of patterns of different symmetry, a systematic exploration of the surface morphology resulting from a variation of laser parameters such as the fluence and number of pulses apart from the pulse separation are required. It is evident that the significance of the wealth of the produced topographies is related to the functionalities they demonstrate and, therefore, appropriate characterization for various properties is necessary.

To account for the capability to intervene in the material’s reorganization process and evaluate the functionalities of the produced topographies, we investigate the LSFL and HSFL formation on stainless steel under a systematic variation of laser parameters that leads to a variety of different morphologies on the micro and nanoscale. Based on the size, shape and hierarchical formation the produced textures were applied over large areas to characterize their wettability properties as well as the cell adhesion behavior and morphology.

## 2. Materials and Methods

Experiments were performed using commercially available, mirror polished samples of 316 stainless steel which were textured with 170 fs laser source emitting at 1026 nm. In these experiments, circularly polarized beams were used and therefore, to produce circularly polarized pulses, a linear polarizer and a quarter-waveplate (QW) plate were used.

**Figure 1:**
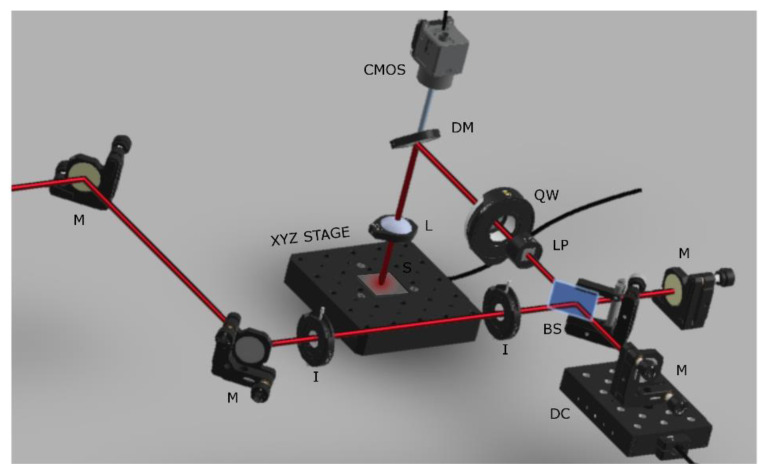
The double pulse setup. Abbreviations; mirror (M), iris (I), beam splitter (BS), linear polarizer (LP), quarter waveplate (QW), dichroic mirror (DM), delay control stage (DC), monitoring camera (CMOS), sample positioning stage (XYZ STAGE).

The experimental setup, shown in Figure 1 consists of irises (I) and mirrors (M) to align and guide the beam, a Michelson interferometer, that is made up of a beam splitter (BS) and a mirror placed in each arm. (All optical components were acquired from Thorlabs Inc., Germany, Europe). The beam is divided into two parts by the beam splitter and then guided into the two arms. One of the two mirrors of the interferometer is controlled by a computer-assisted micrometer displacement controller (DC) which produces an interpulse delay (Δτ) between the two pulses by varying the optical path (L) of the arm. The interpulse delay is given by the expression

(1) $$\Delta \tau \;\left(\mathrm{ps}\right)=2\cdot \frac{\Delta L\left(\mu m\right)}{c}\cdot 10^{6}$$

In Equation (1), c stands for the speed of light, ΔL is the optical path difference of the two beams while the presence of the factor of 2 comes from the fact that the pulse travels two times the displacement of the arm due to the setup geometry. Apart from the pronounced linear polarizer (LP) and a quarter-waveplate (QW), a dichroic mirror (DM) is also included to guide the beam onto the sample (S). A CMOS camera is used to observe the sample surface through a dichroic mirror (DM). The beam is focalized on the sample via a concave lens (L) of focal length f = 20 cm. Finally, the sample was placed on a computer controlled three-axis translational stage (XYZ STAGE). The spot size was calculated to be 60 µm in diameter at 1/e^2^ using a CCD camera placed at focus plane. The experiments were conducted at normal incidence and in ambient air.

The morphologies of the laser-fabricated structures were visualized by a field-emission Scanning Electron Microscope, SEM (JEOL JSM-7000F, JEOL Ltd., Tokyo, Japan). All the measurements of the features of surface structures were performed by a 2D-FFT analysis of the corresponding SEM images using Gwyddion (http://gwyddion.net/, accessed on 8 February 2022), a free and open-source software for SPM (scanning probe microscopy) data visualization and analysis. The frequencies of the induced periodic structures (Λ) were calculated via a 2D-Fourier transformation feature of Gwyddion. The Atomic Force Microscopy (AFM) measurements were carried out using a MultiMode SPM provided by VEECO (Plainview, NY, USA).

### Cell Culture

For cell adhesion testing, murine NIH 3T3 fibroblasts (ATCC, Manassas, VI, USA) and CL57BL/6 bone marrow-derived mesenchymal stem cells (MSCs) (Cyagen, Santa Clara, CA, USA) were used. The stainless steel square surfaces were cleaned by submersion in a 70% ethanol solution and UV sterilization for 20 min. The cells were grown in high glucose (4.5 g/L) Dulbecco’s Modified Eagle Medium (DMEM, Gibco, Grand Island, NY, USA) for NIH 3T3 cells and low glucose (1 g/L) DMEM (Gibco, Grand Island, NY, USA) for MSCs, supplemented with 10% fetal bovine serum (FBS, Gibco, Grand Island, NY, USA) and 1% penicillin/streptomycin (Gibco, Grand Island, NY, USA). Cell were grown at 37 °C in a 5% CO_2_ atmosphere. Fresh medium was provided every 2 days until the desired timepoints (3 and 5 days) were reached. SEM was used to visualize cell morphology and attachment on the structured and non-structured stainless steel samples. At each desired timepoint, the medium was removed, followed by two 7 min washes with 1×PBS on ice. Samples were fixed with 2.5% glutaraldehyde (GDA, Sigma-Aldrich, St Louis, MO, USA) in 0.1 M Sodium Cacodylate Buffer (SCB) for 30 min and two 7 min washes with 0.1 M SCB were performed. Dehydration was achieved with 7 min immersions of the samples in ethanol gradients (30%, 50%, 70%, 90%, 100%). Samples were dried using increasing concentrations of hexamethyl-disilazine (HDMS, Sigma-Aldrich, St Louis, MO, USA) in ethanol (30%, 50%, 70%, 3 × 100%) and complete evaporation of HDMS was achieved by leaving them in a chemical hood overnight. Prior to SEM imaging (JEOL JSM-6390 LV, JEOL Ltd., Tokyo, Japan), all samples were sputter-coated with a 12 nm-thick gold layer using a Baltec SCD 050 instrument (BAL-TEC AG, Balzers, Liechtenstein).

## 3. Results and Discussion

A parametric study has been performed to analyze the LSFL and HSFL formation on stainless steel under a systematic change of laser parameters that leads to a variety of different morphologies at the micro and nanoscale. The objective is, firstly, to tailor the morphology through a control of the laser parameters such as the fluence, pulses per spot and, predominantly, the pulse separation, and secondly, to evaluate the impact of the different patterns on the wetting and cell adhesion properties. Based on the results of parametric studies presented in previous reports [18,33], in this work, two different interpulse delay (Δτ) values in the picosecond regime were chosen to be investigated thoroughly in order to generate structures with various spatial characteristics; Δτ = 5 ps to study the hierarchical 2D morphology consisting of HSFL and LSFL [18] and Δτ = 20 ps to explore the 2D-HSFL formation [26].

### 3.1. Controlling the Morphology

#### Impact of Interpulse Delay

**Figure 2:**
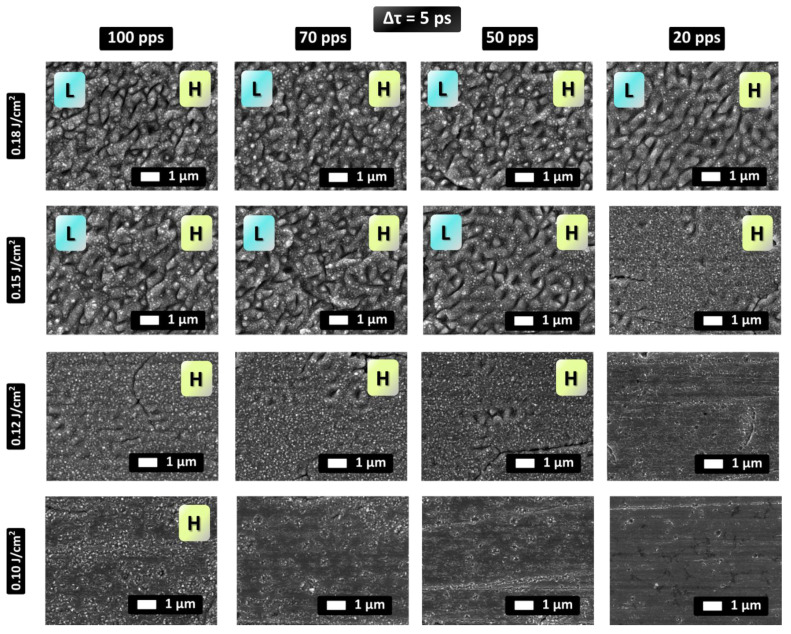
SEM images of stainless steel surfaces processed with double pulses with interpulse delay Δτ = 5 ps for different values of fluence and pps. L and/or H indicate whether LSFL and/or HSFL features, respectively, appear on the topographies.

**Figure 3:**
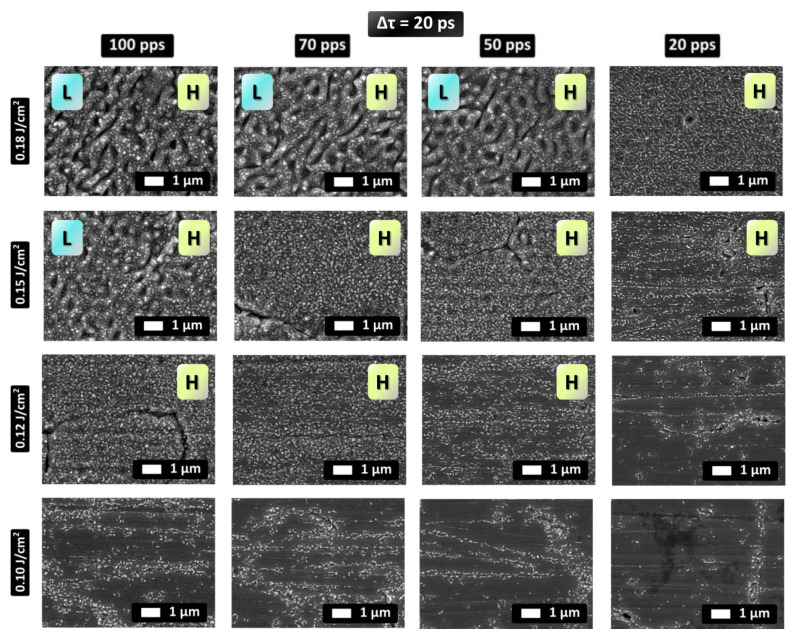
SEM images of stainless steel surfaces processed with double pulses with interpulse delay Δτ = 20 ps for different values of fluence and pps. L and/or H indicate whether LSFL and/or HSFL features, respectively, appear on the topographies.

Surface morphologies have been obtained by increasing the laser fluence in a range between F = 0.1 J/cm^2^ and F = 0.18 J/cm^2^, and for pulses per spot (pps) values varying from pps = 20 (5 mm/s) to pps = 100 (20 mm/s) are illustrated in Figure 2 (Δτ = 5 ps) and Figure 3 (Δτ = 20 ps). The induced patterns contain 2D-LSFL and HSFL structures.

Upon a variation of laser parameters and appropriate combination of the fluence and pps values, various patterns decorated with LSFL and HSFL features (denoted with ‘L’ and ‘H’), respectively, are produced and illustrated in Figure 2 (Δτ = 5 ps). Experimental observations show that the possibility of fabricating L-type structures increases at higher energy doses and fluence values. For example, at Φ = 0.18 J/cm^2^, random 2D–LSFL are the dominant structures whereas at Φ = 0.12 J/cm^2^, 2D-HSFL are formed on the surface. On the other hand, the evolution of surface topographies shows (Figure 2) that for the highest dose, at Φ = 0.18 J/cm^2^, 2D-LSFL as well as random HSFL and a small population of nanodots are fabricated. These particular 2D-LSFL structures appear to become less pronounced as the dose decreases. Furthermore, at this fluence, HSFL structures preserve their randomness while the nanodots population decreases at decreasing pps. At a slightly smaller fluence (Φ = 0.15 J/cm^2^) a similar structure evolution is observed; however, LSFL structures become less pronounced at decreasing pps; finally, at pps = 20, 2D-HSFL structures dominate on the surface and LSFL have almost disappeared. At an even lower fluence, Φ = 0.12 J/cm^2^, LSFL features are almost absent while random 2D-HSFL and a large population of nanodots are observed at relatively high pps value. By contrast, as pps further decreases, 2D-HSFL are formed while nanodots are getting less pronounced. When pps = 20 only shallow random 2D-HSFL structures are present. Lastly for fluence Φ = 0.1 J/cm^2^ and pps = 100, random 2D-HSFL features are formed which gradually disappear as pps number drops. Interestingly, at Φ = 0.1 J/cm^2^ and pps = 20, only a small number of features are observed on the surface around holes and cracks highlighting that these parameters are close to the HSFL formation threshold.

SEM images in Figure 3 illustrate the types of patterns which are produced if the pulse separation is increased to Δτ = 20 ps. According to the experimental observations, the L-type structures dominate at higher energy doses and larger fluences. For example, at Φ = 0.18 J/cm^2^ random 2D–LSFL are the dominant structures whereas at Φ = 0.15 J/cm^2^ HSFL are formed on the surface.

In a more detailed description of the evolution of the surface topographies as a function of the fluence and the energy dose (Figure 3), it is shown that for the highest pps, at Φ = 0.18 J/cm^2^, 2D-LSFL together with random HSFL and a small population of nanodots are formed. On the other hand, at the same fluence and at decreasing pps values, 2D-LSFL become less pronounced and at pps = 20, only 2D-HSFL features are present. At Φ = 0.15 J/cm^2^ and pps =100, 2D-LSFL and 2D-HSFL are formed simultaneously while nanodots and 2D- HSFL dominate for lower pps values. At lower values dose values of the nanodots and HSFL are getting less and less pronounced until they vanish at the lowest fluence used in the experiments, Φ = 0.1 J/cm^2^ and pps = 20.

A comparison between the patterns for the two delays (Δτ = 5 ps and Δτ = 20 ps) shows that both L- and H-type of structures are produced in both cases; however, the pps threshold value that leads to the onset of their formation increases at longer pulse separation. More specifically, random 2D-LSFL structures occur at higher energy fluence (Φ ≥ 0.15 J/cm^2^) while random 2D-HSFL and nanodots occur for lower energy fluence (Φ ≤ 0.12 J/cm^2^). Nonetheless, LSFL formation threshold seems to be lower for the case when Δτ = 5 ps (Φ = 0.15 J/cm^2^, pps = 50) than in the case when Δτ = 20 ps (Φ = 0.18 J/cm^2^, pps = 50). Moreover, 2D-HSFL are more pronounced at 5 ps delay and nanodots are more pronounced at a 20 ps delay. With respect to the role of the fluence, for both interpulse delays, LSFL occur at higher fluences but for different energy dose thresholds. In the case of 5 ps delay, LSFL are present at fluences from Φ = 0.15 J/cm^2^ to Φ = 0.18 J/cm^2^ at almost all pps values while in the 20 ps delay case, LSFL occur mainly at the highest fluence Φ = 0.18 J/cm^2^. In the case of 5 ps delay, 2D HSFL are not so clear and occur together with LSFL while in case of 20 ps delay 2D-HSFL and nanodots cover the total surface at Φ ≤ 0.12 J/cm^2^.

**Figure 4:**
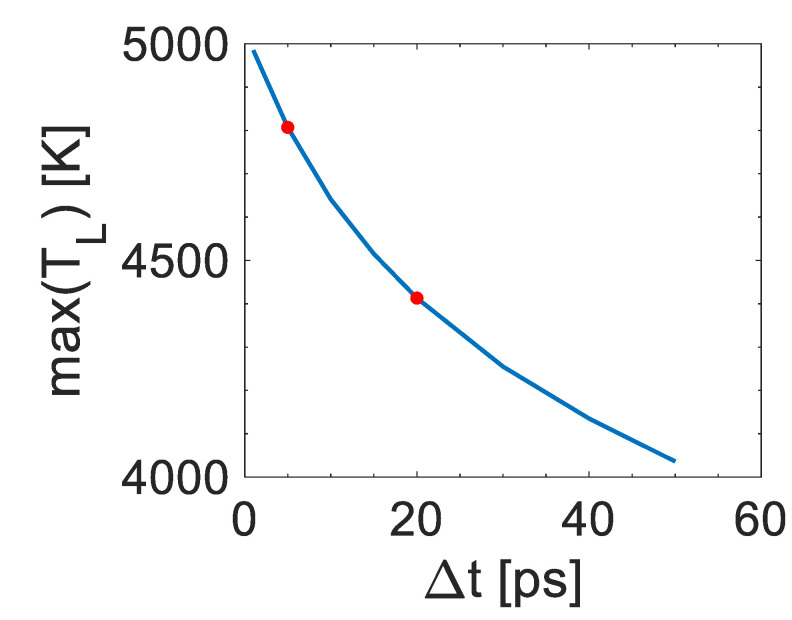
Maximum Lattice temperature as a function of the interpulse delay.

To emphasize the impact of the pulse separation on the different patterns that are induced, simulations have been conducted to predict the thermal response of the irradiated material. A Two Temperature Model was employed to simulate the ultrafast dynamics of the system. A detailed description of the model and the values of the thermophysical parameters are presented in Reference [34]. Theoretical results (Figure 4) show that the maximum lattice temperature attained for Δt = 5 ps is higher than that for Δt = 20 ps for double pulses of *F_total_* = 0.15 J/cm^2^ (*F_total_* corresponds to the total fluence of the double pulse). It is noted that for the sake of simplicity, the simulations have been performed for flat areas, while the approach can be generalized at higher energy doses that are expected to lead to even higher variance between the calculated temperatures for the two pulse separation values. Furthermore, higher energy doses increase the roughness of the irradiated surface and induce LIPSS formations leading to an increase of the absorbed energy as it is known that sub-wavelength surface structures significantly enhance absorptance through plasmonic absorption. Therefore, as patterns for interpulse delay Δt = 5 ps and Δt = 20 ps are characterized with different roughness and features (height, shape, etc.), the features of the electromagnetic modes that are excited and the resulting form of the absorbed spatial energy distribution are expected to lead to the formation of distinctly different structures.

**Figure 5:**
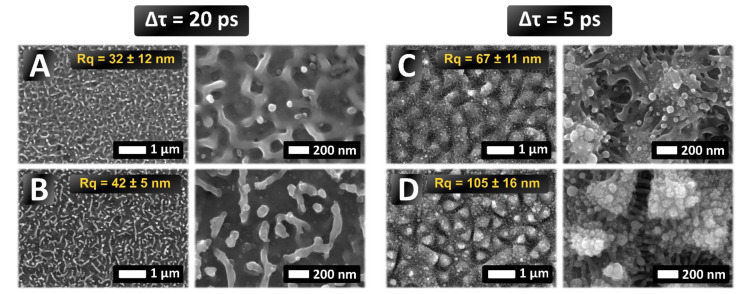
SEM images of topographies obtained after optimization of process parameters. Process parameters: (**A**): Φ = 0.16 J/cm^2^, pps = 20, (**Β**): Φ = 0.14 J/cm^2^, pps = 100, (**C**): Φ = 0.18 J/cm^2^, pps = 15, (**D**): Φ = 0.18 J/cm^2^, pps = 60.

As a result of an extensive parametric study surfaces with distinct characteristics in terms of average roughness value, Rq, symmetry and spatial period were produced. The textured surface spanned over a 4 × 4 mm^2^ area while the processing time varied between 80 s (Figure 5C) and 320 s (Figure 5B). It is noted that the average roughness values were obtained from AFM measurements, and they correspond to the root mean square (rms) for a set of values obtained in a patterned area of (minimum) size equal to 5 × 5 μm^2^. Four different surfaces are presented, shown in Figure 5 comprising solely HSFL (Figure 5A,B) or a mixture of HSFL and LSFL (Figure 5C,D) which are ordered according to the surface roughness. Different magnifications were utilized in each case to visualize features in the micro and nanoscale. As shown in the previous section, results indicate that at Δτ = 20 ps, HSFL-type structures dominate the topographies that are produced for the range of fluence values used in the experiments. A more thorough analysis of the morphological features of the HSFL structures formed at various fluences and pps show that for Φ = 0.16 J/cm^2^ and pps = 20, HSFL with random direction are formed together with a few nanodots (Figure 5A). The average roughness of the produced morphology is Rq = 32 ± 12 nm while the structure height was measured to be equal to h = 55 ± 8 nm. The average distance ρ between neighboring HSFL protrusions has been measured (ρ = 206 ± 122 nm) while their width is equal to W_HSFL_ = 66 ± 10 nm. The measured value of the diameter δ of the dots is δ_dot_ = 53 ± 13 nm. It is noted that the average values of ρ and δ_dot_ were calculated from a sample of at least 25 measurements in the SEM images. Results shown in Figure 5B indicate that, at higher doses (Φ = 0.14 J/cm^2^, pps = 100), random HSFL are formed together with dots. The average roughness of the surface pattern is Rq = 42 ± 5 nm which appears to be slightly larger (but within the experimental error) compared to the morphology in Figure 5A and the structure height is measured to be equal to h = 66 ± 30 nm. On the other hand, the diameter of the dots is δ_dot_ = 41 ± 13 nm and the width of the HSFL W_HSFL_ = 62 ± 11 nm. The average distance of the structures is ρ = 222 ± 60 nm which is comparable with the value measured in the previous case. Thus, the morphologies in Figure 5A,B comprise similar spatial characteristics (ρ, δ_dot,_ W_HSFL_) while the aspect ratio of the structures differs.

By contrast, the simultaneous formation of HSFL and LSFL that can be achieved for Δτ = 5 ps (Figure 5C,D) allows to further increase the surface roughness while at the same time maintaining the characteristic nano-decoration of HSFL-like structures and dots. In Figure 5C the LSFL structures are formed on the surface together with a HSFL which seem to be formed in between the LSFL structures.

The 2D-LSFL average distance here is ρ = 890 ± 35 nm, the surface roughness Rq = 67 ± 11 nm and the height is measured to be equal to h = 146 ± 53 nm. At higher energy doses (Figure 5D), the pattern consists of an inhomogeneous hierarchical 2D-LSFL morphology, and more specifically HSFL and dots are formed between and on top of LSFL, respectively. The measured period of the 2D-LSFL is Λ_LSFL_ = 867 ± 25 nm. The average period of HSFL structures is Λ_HSFL_ = 109 ± 10 nm whilst the average diameter of dots is δ_dot_ = 44 ± 14 nm. The average roughness here is Rq = 105 ± 16 nm and the height is h = 264 ± 122 nm for Figure 5D. The height of the 2D-LSFL structures is in agreement with observations in previous reports [20].

### 3.2. Wetting Properties

**Figure 6:**
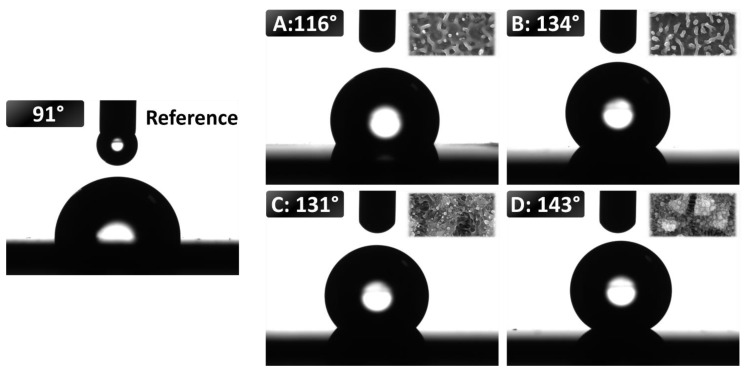
Contact angle measurements of stainless steel surface textured as shown in Figure 5. 4 μL drops were used. The letter (**A**–**D**) indicate the type of the surface as declared in Figure 5. A small indicative SEM image of the relevant structures is show on the top right of every image.

Contact angle (CA) measurements were carried out to characterize the wetting properties of structures formed on larger areas (1 × 1 cm^2^) (Figure 6). It is noted that CA measurements were performed on the samples one month after the irradiation. That timeframe is considered to be sufficient for the processed area to exhibit a conclusive wetting behavior [35]. Figure 6 shows the results of the measurements while the insets (SEM figures) are included to facilitate the correlation between Figure 5 and Figure 6. The unprocessed surface (Figure 6, ’Reference’) has a contact angle of 91° whilst all textured surfaces exhibit a hydrophobic behavior. A detailed analysis indicates that random HSFL (Figure 6A) exhibit a 116° contact angle while a mixture of HSFL with dots (Figure 6Β) yields a 134° contact angle; on the other hand, shallow LSFL and HSFL (Figure 6C) have a 131° contact angle while a well-developed random 2D-LSFL has a 143° contact angle (Figure 6D). A correlation between the surface roughness and the contact angle can be established which confirms that topographies of various roughness and complexity exhibit different wetting properties [36]. Our results show that for structures of similar symmetry and average size, a roughness increase leads to higher contact angle values. More specifically, for HSFL, a lower roughness topography (Rq = 32 ± 12 nm, Figure 6A) exhibits a smaller contact angle compared to a pattern of higher roughness (for Rq = 42 ± 12 nm, Figure 6B). Similarly, for LSFL structures, we observe an increase of the surface roughness from Rq = 67 ± 11 nm (Figure 6C) to Rq = 105 ± 16 nm (Figure 6D) results into an increase of the contact angle. By contrast, a comparison of the wetting properties of topographies that have different symmetry and hierarchical formation (LSFL and HSFL structures) does not lead to conclusive arguments and therefore more investigation is required.

### 3.3. Nanostructure’s Cell Adhesion Properties

Four distinctly different types of morphologies (Figure 5A–D) were investigated for their cell adhesion and proliferation properties. Two types of cells were used for testing: (a) NIH 3T3 murine fibroblasts and (b) murine bone marrow-derived mesenchymal stem cells (MSCs) that were grown in a medium maintaining them in an undifferentiated state. These two cell types selected as fibroblasts play an important role in wound healing and regeneration, while mesenchymal stem cells are multipotent and previous research has demonstrated that fs-laser processing of Ti was able to induce them towards osteogenic differentiation [37].

#### 3.3.1. NIH 3T3 Cell Behavior

As shown in Figure 6, the contact angle measurements indicate that the different topographies are more hydrophobic compared to the flat steel surface. Surface hydrophobicity is known to affect cellular behavior at early timepoints, as the repellence of a surface can inhibit cellular adhesion. After 3 days–and especially after 5 days–, protein adsorption onto the surface, either from the proteins that are present in the serum or from proteins that are produced by the cells, alters the environment and makes it more hydrophilic and cell friendly. As such, at the two timepoints tested within this study, we have focused on the effect of the topography on cell behavior.

**Figure 7:**
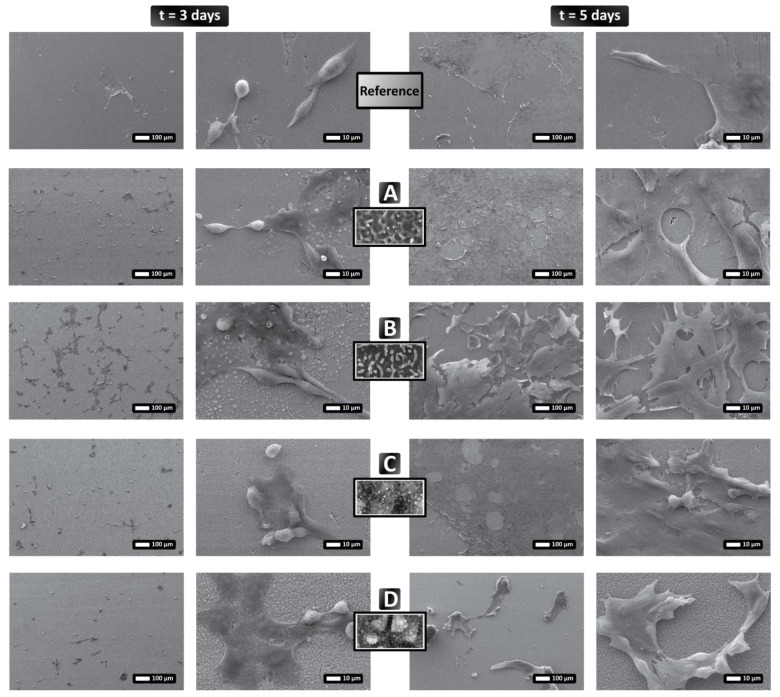
Growth of NIH 3T3 fibroblast cells on patterned stainless steel surfaces. Fibroblasts were cultured for 3 and 5 days on flat stainless steel surfaces and four different laser-induced surface structures. The cells adhered differently to the different surfaces, indicating that the topography influences cellular behavior. The letters (**A**–**D**) indicate the type of surface as declared in Figure 5, while “Reference” refers to the flat stainless steel surface. The SEM images show low and high magnifications of the samples (scale bars of 100 μm and 10μm, respectively), while the insets indicate the relevant underlying surface structure.

Results show (Figure 7) that NIH 3T3 cells appear to interact differently with the different patterned surfaces. More specifically, after 3 days, cells adhere better to the pattern in Figure 7B (textured surface contains a mixture of HSFL and nanodots), where a larger number of more flattened, well-adhered cells can clearly be seen. By contrast, in all other areas, the number of cells is substantially smaller and there is a mixture of globular and flattened cells, which indicates weaker adhesion. After 5 days, the cells appear to have proliferated; however there is different behavior depending on the textured area. In the areas shown in Figure 7A (pattern contains random HSFL) and C (textured area comprises a combination of LSFL and HSFL structures), the cells have adhered efficiently to the surface and created a “carpet” (similar to the flat steel surface in Figure 7, Reference). On the surface illustrated in Figure 7D (covered with random 2D-LSFLstructures), the cells have grown in clumps/aggregates and have not spread on the whole surface, while on the surface shown in Figure 7B, the cells have been distributed similarly to Figure 7A,C, but seem to be substantially less attached to the surface.

#### 3.3.2. Mesenchymal Stem Cell Behavior

**Figure 8:**
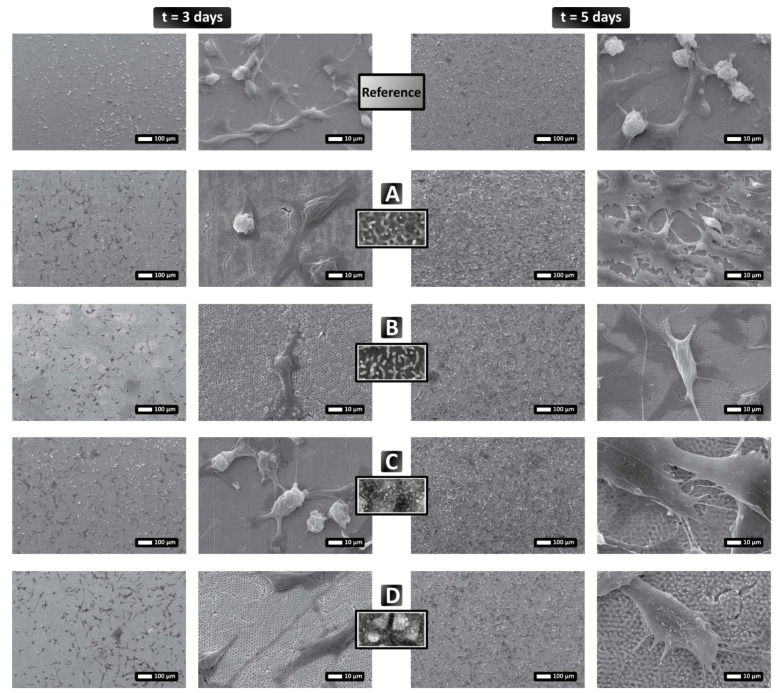
Growth of mesenchymal stem cells (MSCs) on patterned stainless steel surfaces. MSCs were cultured for 3 and 5 days on flat stainless steel surfaces and four different laser-induced surface structures. The cells have adhered to all the surfaces and have proliferated much more significantly compared to the fibroblasts shown in Figure 7. The letters (**A**–**D**) indicate the type of surface as declared in Figure 5, while “Reference” refers to the flat stainless steel surface. The SEM images show low and high magnifications of the samples (scale bars of 100 μm and 10 μm, respectively), while the insets indicate the relevant underlying surface structure.

Contrary to the behavior displayed on the various topographies by the fibroblasts in Figure 7, MSCs respond differently to the same patterns, as they adhere and proliferate on all areas (Figure 8). After 3 days, there are fewer cells on the pattern shown in Figure 8B (textured surface contains a mixture of HSFL and nanodots); however, after 5 days in culture, the cells have proliferated and almost completely covered all areas, regardless of the underlying pattern. As such, we can conclude that MSC attachment is good on all surfaces, while the presence of filopodia and lamellipodia is clearly observable (Figure 8A–D.) It is noted that the textured area comprises: (i) random HSFL (Figure 8A), (ii) a mixture of HSFL and nanodots (Figure 8B), (iii) a combination of LSFL and HSFL structures (Figure 8C), (iv) random 2D-LSFLstructures (Figure 8D).

#### 3.3.3. Discussion of Nanostructure’s Cell Adhesion Properties

A general conclusion from the above results is that structures with different spatial characteristics (random 2D-LSFL and random 2D-HSFL) can exhibit variable cell adhesion properties. Cellular behavior changes depending on the underlying structure, as well as the type of cell used. For the surfaces tested in this study, fibroblasts display greater variability in their attachment profiles, while undifferentiated mesenchymal stem cells have a much higher affinity for all structures and can almost completely saturate them after 5 days in culture. This could be attributed to the different physiological function of the two types of cells: fibroblasts are more adapted to respond to smooth and soft surrounding surfaces, while MSCs, due to their multipotent nature, are more adaptable and can respond to external stimuli (such as surface roughness or different material hardness) to differentiate into cells of different lineages. As MSCs have the ability of differentiating into osteoblasts, it would explain their ability to interact more strongly with the hard surfaces provided by the stainless steel, compared to the fibroblasts.

By comparing the cellular behavior of one type of cell on the different topographies, as well as the two different types of cells on the same topography, we can potentially utilize these results towards the fabrication of tailored surfaces for the development of functional implants. Tissue repair is an extremely complex process and, through exploring different methodologies for tissue engineering applications, we can create functional surfaces where certain types of cells can adhere to promote repair, while other types of cells are deterred, thus inhibiting adverse reactions such as fibrosis.

## 4. Conclusions

An experimental study has been performed to investigate the effect of the interpulse delay of double pulses on the features of induced LIPSS on stainless steel surfaces. An analysis of the experimental results and comparison with observations from previous studies demonstrates that an interpulse separation equal to Δτ = 5 ps favors the formation of 2D-LSFL, while longer delays (Δτ = 20 ps) are more suitable for the fabrication of 2D-HSFL structures. The produced morphologies were characterized for their wetting and cell adhesion properties and a correlation emerges between the surface roughness and the wetting behavior of the surface. On the other hand, cell culture experiments unveil the need to introduce a more complex scenario in order to interpret how cell adhesion is affected from both the surface nano-roughness and the symmetry and the size of the features. Certainly, this hypothesis requires further experimental investigation and could potentially pave the way for the fabrication of cell specific surfaces. The presented results are aimed at enhancing our knowledge of matter rearrangement and fabrication of 2D-LIPSS with temporarily separated fs pulses on the nanoscale, while at the same time the methodology can be employed to establish a connection between the surface topography features and novel material properties depending on the texture’s geometry.

## Data Availability

Not applicable.
